# Supplementary figures and images for: Oxaliplatin resistance in colorectal cancer enhances TRAIL sensitivity via death receptor 4 upregulation and lipid raft localization
Source: eLife. 2021 Aug 3;10:e67750. doi: 10.7554/eLife.67750 (PMC8331188; doi:10.7554/eLife.67750)

Figure 2 Source Data

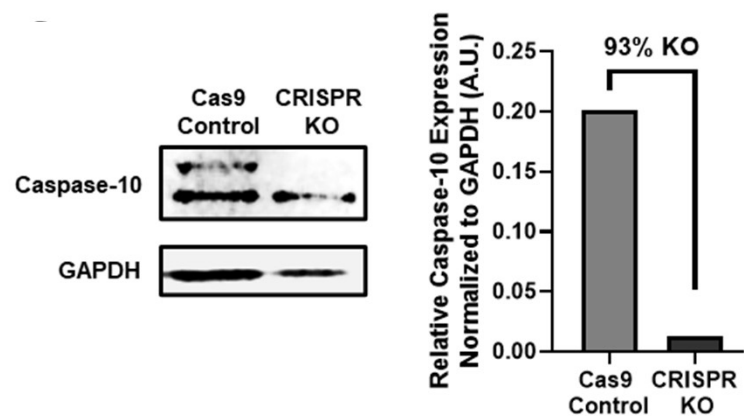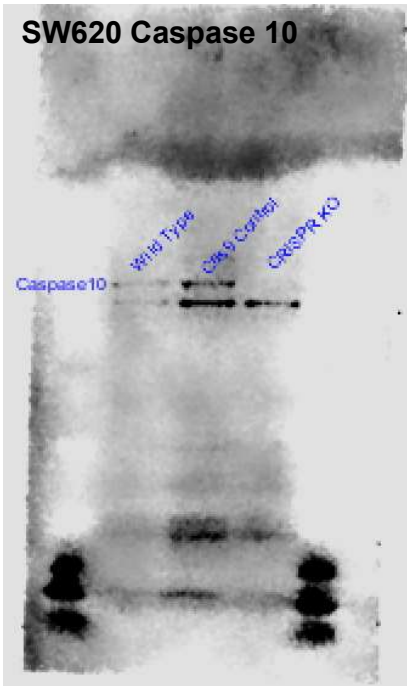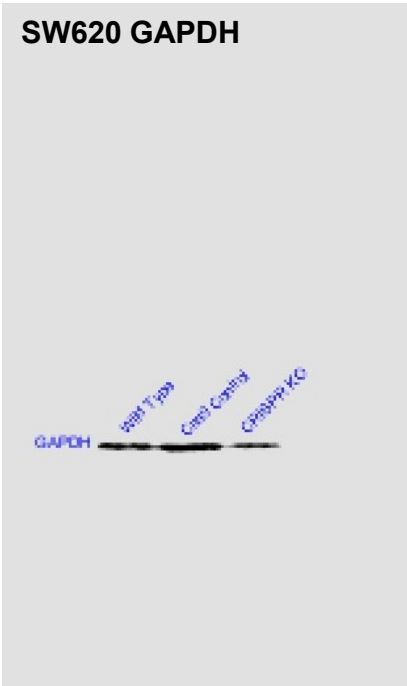

Supplement: Figure 2—source data 3. [file elife-67750-fig2-data3.zip › Figure 2-source data 3 (blots)/Figure 2-source data 3.pdf]

Figure 3 Source Data

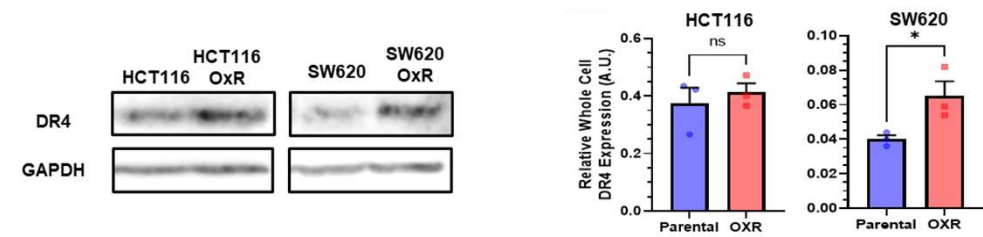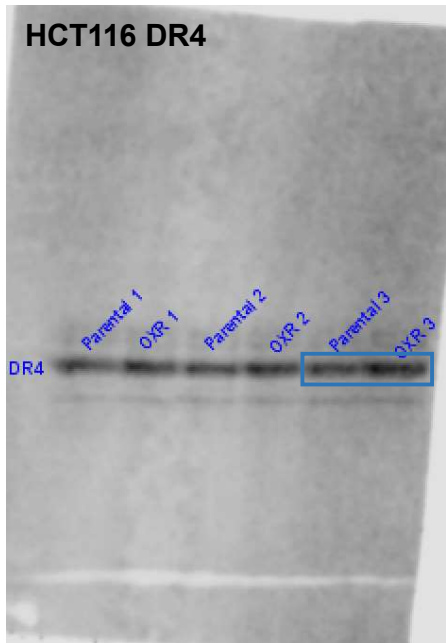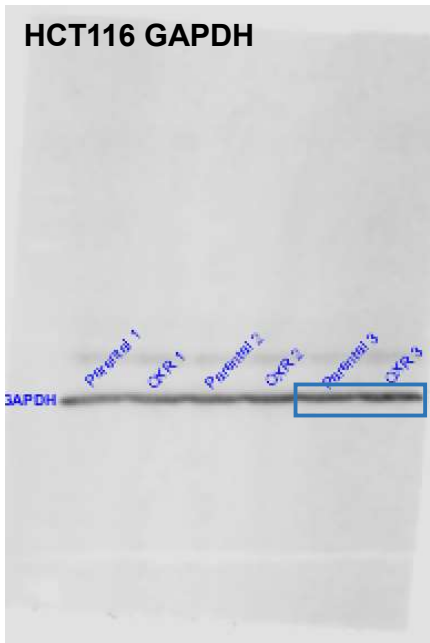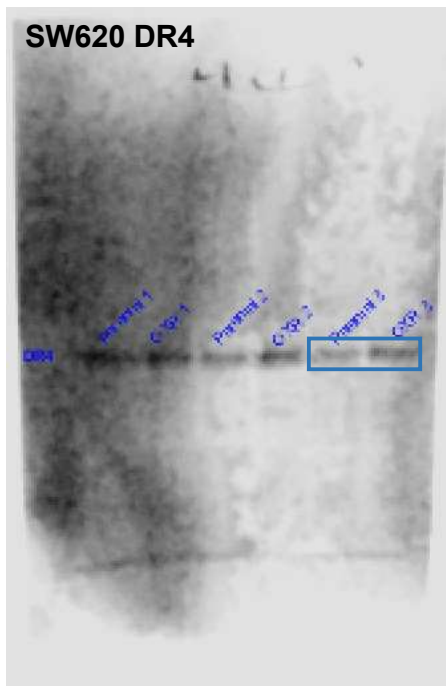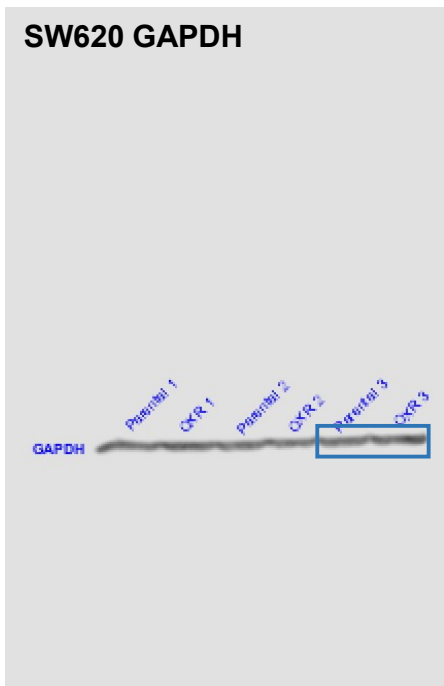

Supplement: Figure 3—source data 2. [file elife-67750-fig3-data2.zip › Figure 3-source data 2 (blots)/Figure 3-source data 2.pdf]

Figure 3 Supplement 5 Source Data

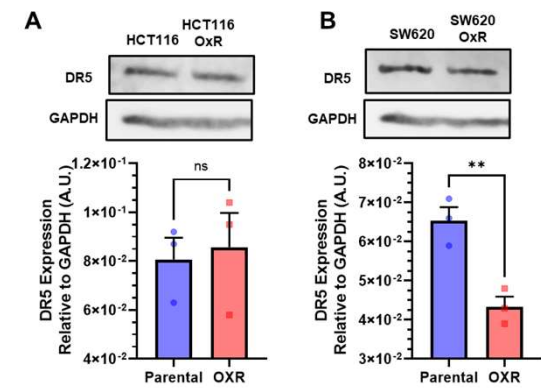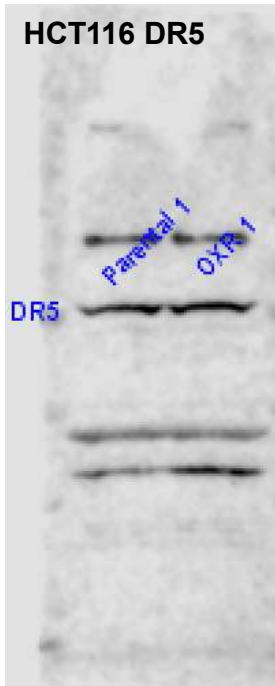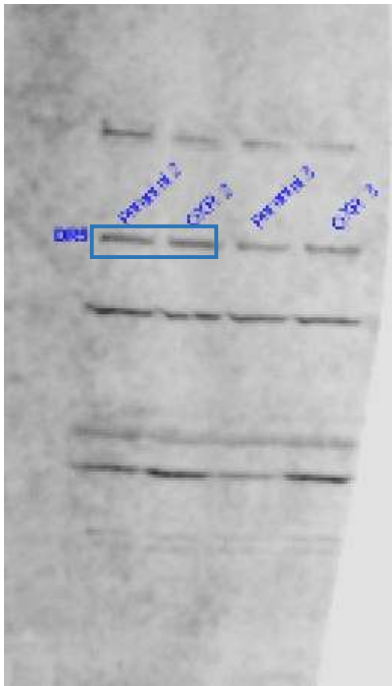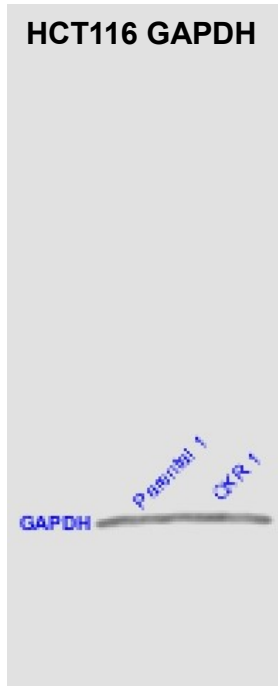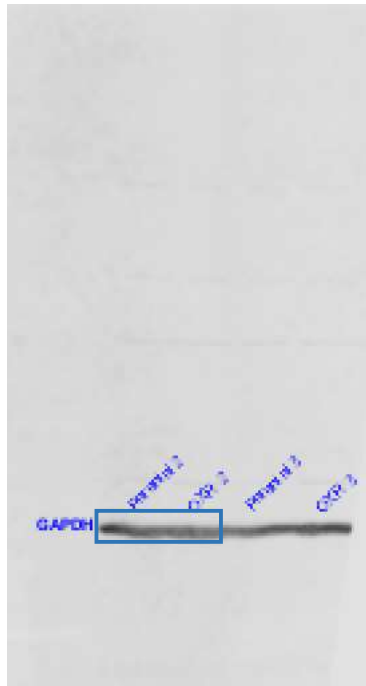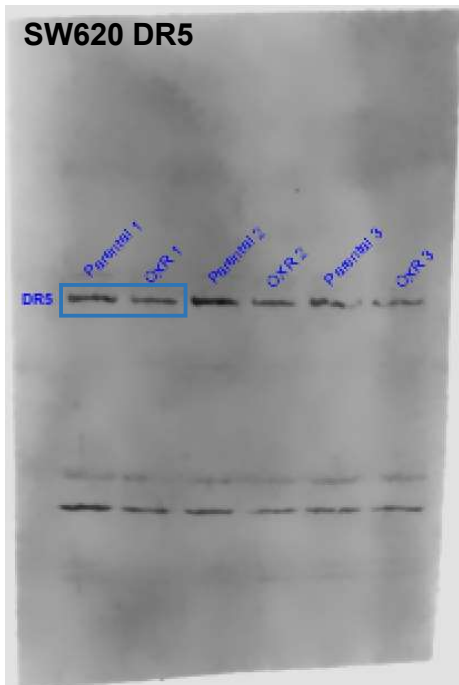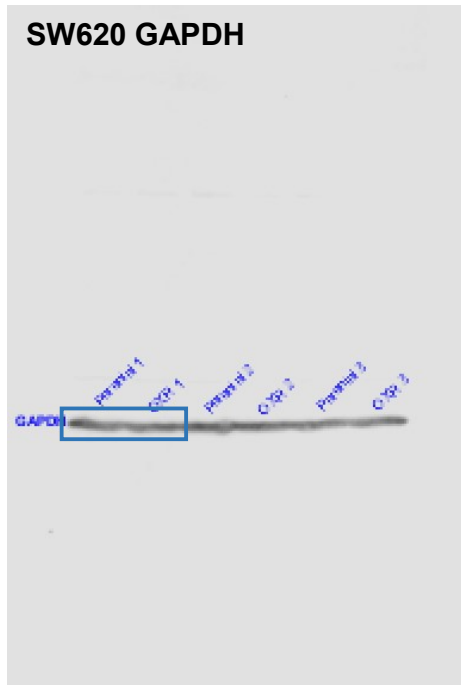

Supplement: Figure 3—figure supplement 5—source data 2. [file elife-67750-fig3-figsupp5-data2.zip › Figure 3-figure supplement 5-source data 2 (blots)/Figure 3-figure supplement 5-source data 2.pdf]

Figure 4 Source Data

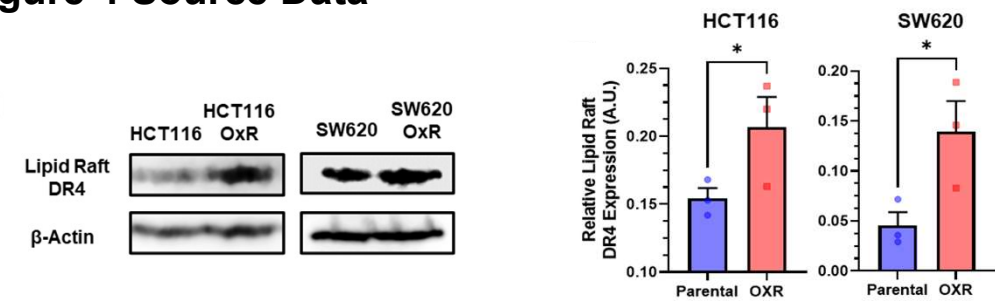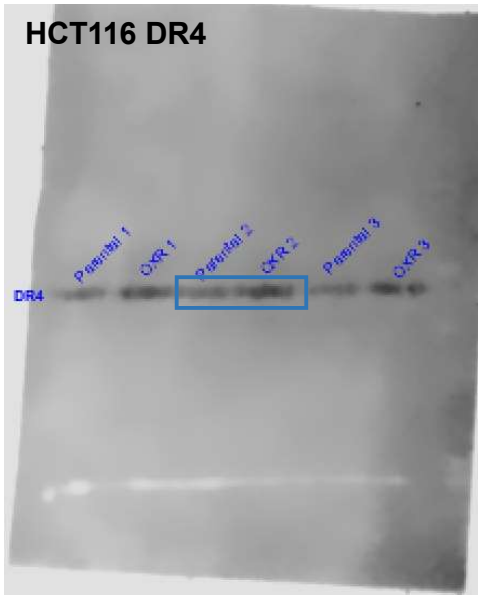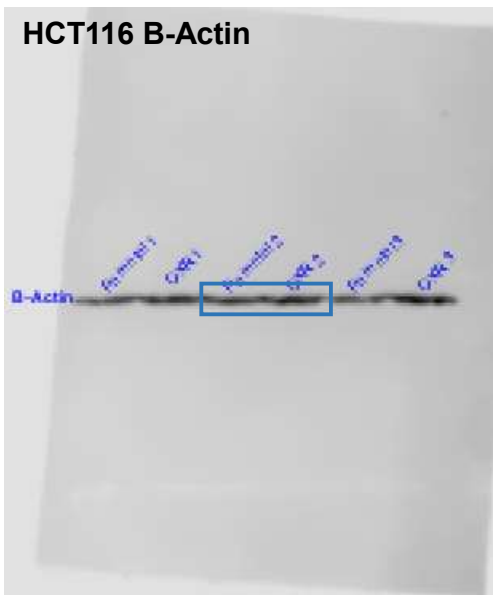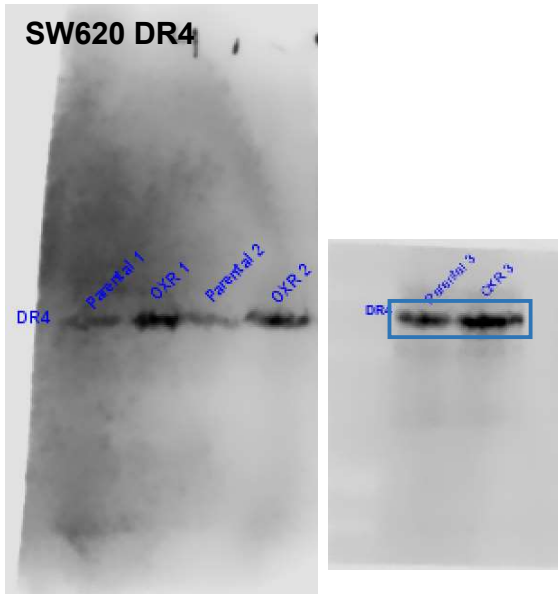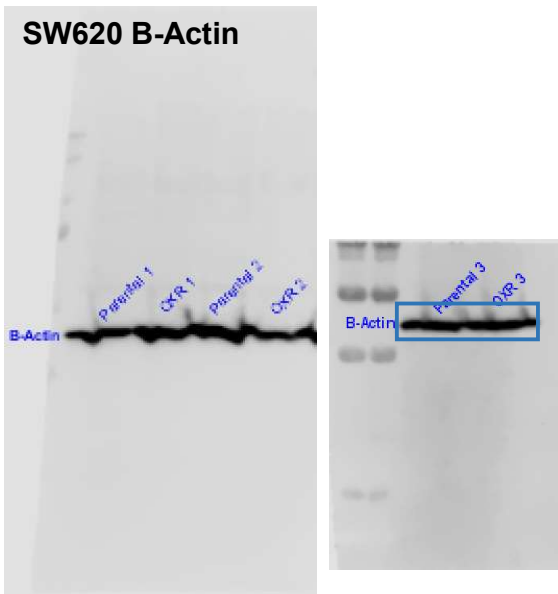

Supplement: Figure 4—source data 3. [file elife-67750-fig4-data3.zip › Figure 4-source data 3 (blots)/Figure 4-soure data 3.pdf]

Figure 4 Supplement 2 Source Data

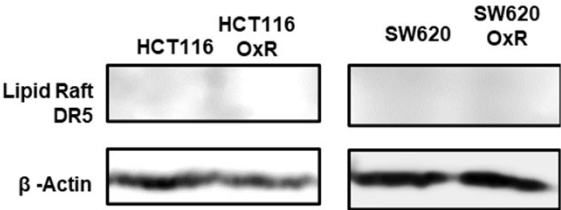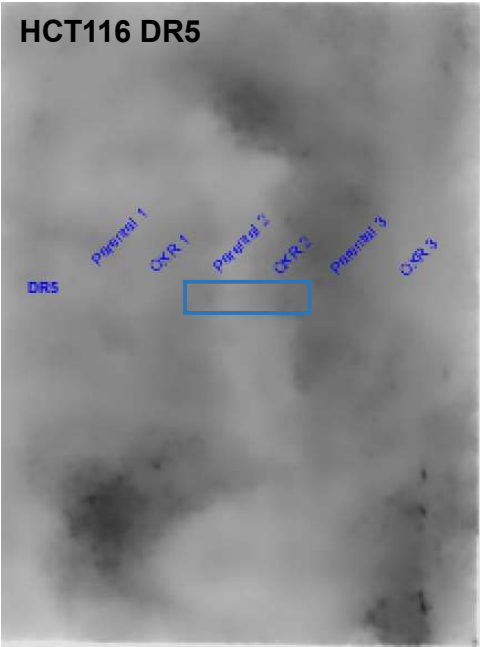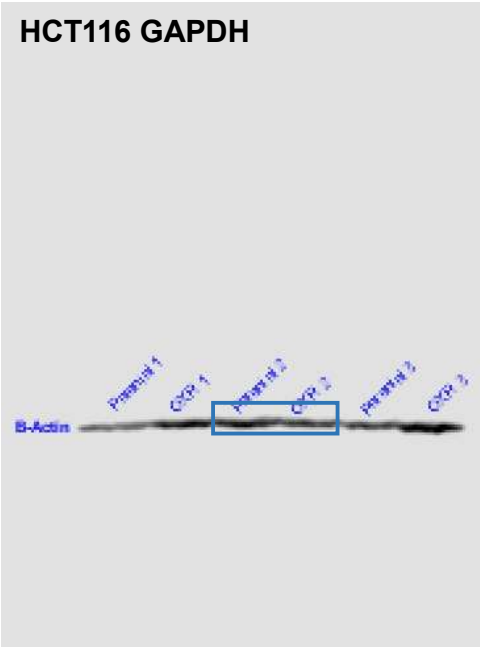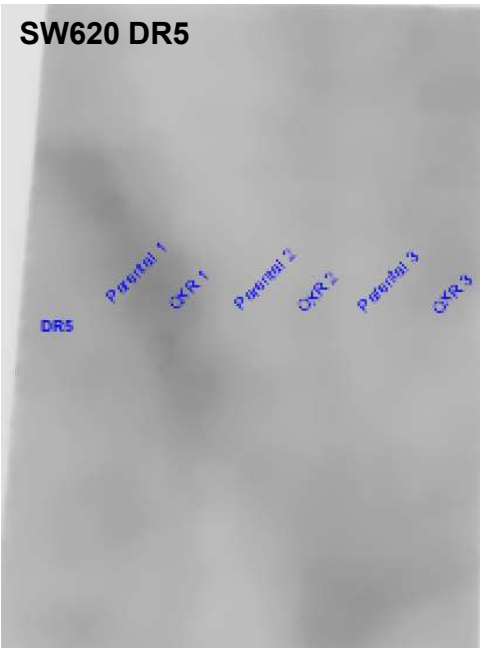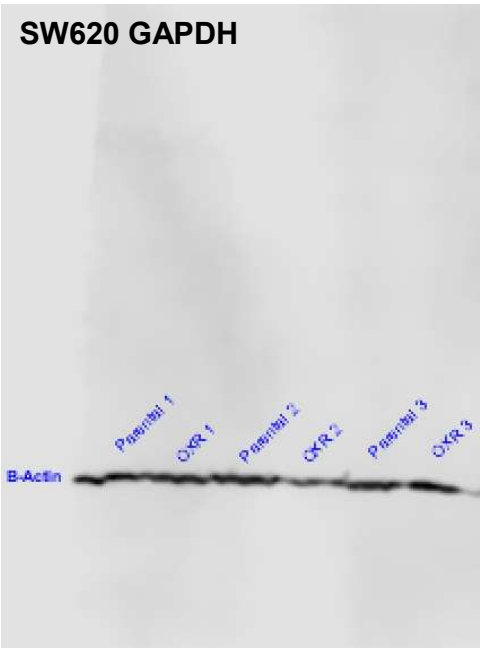

Supplement: Figure 4—figure supplement 2—source data 3. [file elife-67750-fig4-figsupp2-data3.zip › Figure 4-figure supplement 2-source data 3 (blots)/Figure 4-figure supplement 2-source data 3.pdf]

Figure 6 Source Data

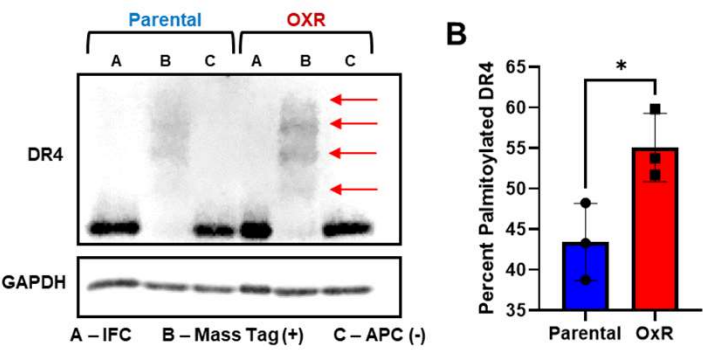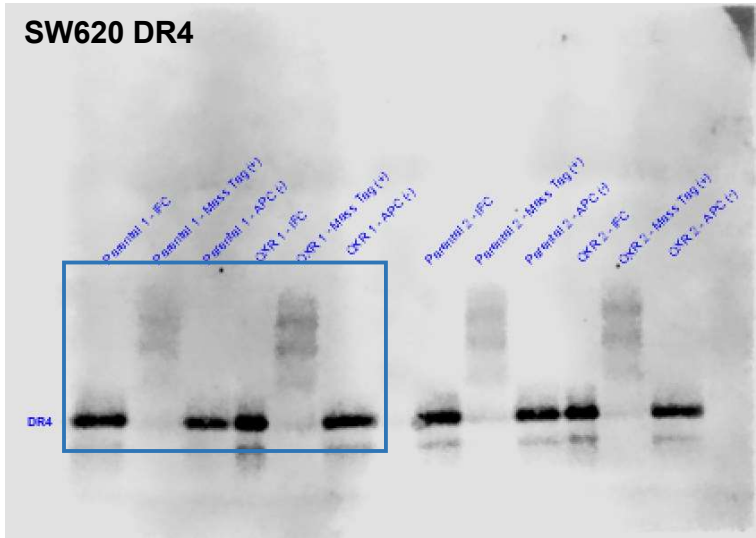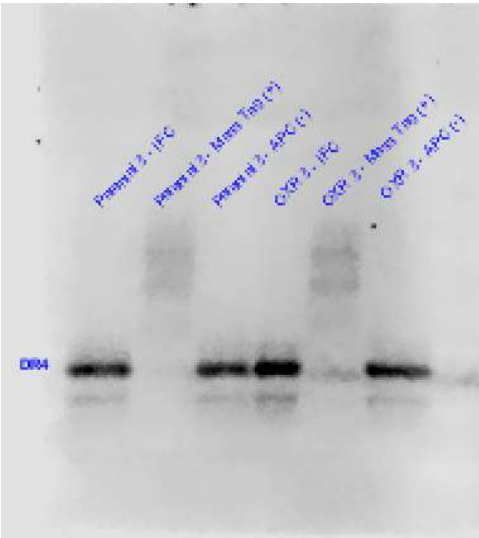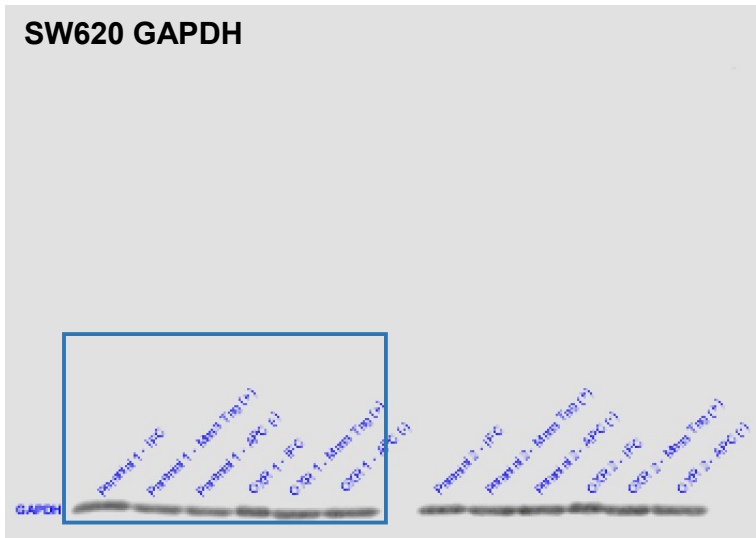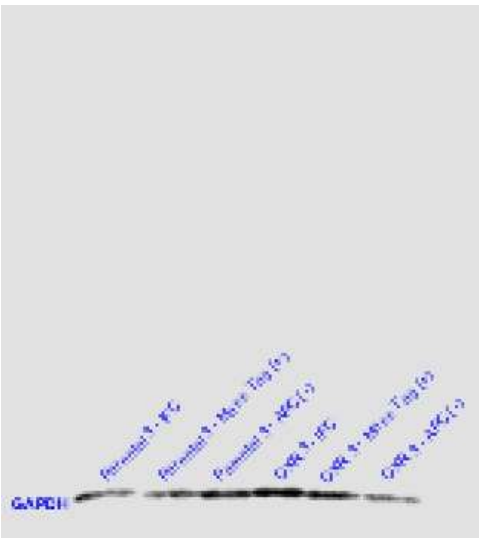

Supplement: Figure 6—source data 3. [file elife-67750-fig6-data3.zip › Figure 6-source data 3 (blots)/Figure 6-source data 3.pdf]
