# Supplementary material for: Oxaliplatin resistance in colorectal cancer enhances TRAIL sensitivity via death receptor 4 upregulation and lipid raft localization
Source: eLife. 2021 Aug 3;10:e67750. doi: 10.7554/eLife.67750 (PMC8331188; doi:10.7554/eLife.67750)
Supplement: Supplementary file 1. — Significance was determined if chi-squared T(x) sample > chi-squared T(x) background. [file elife-67750-supp1.docx]

| **Cell line** | **Stain** | **Chi-Squared T(x)**  **Background**  **(OxR vs Parental)** | **Chi-Squared T(x)**  **Sample**  **(OxR vs Parental)** | **Significance** |
| --- | --- | --- | --- | --- |
| SW620 | DR4 | 43.54 | 106.74 | **Yes** |
| SW620 | DR5 | 43.54 | 307.20 | **Yes** |
| SW620 | DcR1 | 43.54 | 11.70 | **No** |
| SW620 | DcR2 | 43.54 | 35.37 | **No** |
| SW620 | FasR | 2.95 | 271.82 | **Yes** |
| HCT116 | DR4 | 443.32 | 1182.10 | **Yes** |
| HCT116 | DR5 | 443.32 | 95.20 | **No** |
| HCT116 | DcR1 | 443.32 | 53.28 | **No** |
| HCT116 | DcR2 | 443.32 | 11.32 | **No** |
| SW480 | DR4 | 88.47 | 26.34 | **No** |
| SW480 | DR5 | 88.47 | 17.46 | **No** |
| SW480 | DcR1 | 88.47 | 46.67 | **No** |
| SW480 | DcR2 | 88.47 | 99.23 | **Yes** |
| HT29 | DR4 | 54.33 | 76.58 | **Yes** |
| HT29 | DR5 | 54.33 | 29.15 | **No** |
| HT29 | DcR1 | 54.33 | 95.81 | **Yes** |
| HT29 | DcR2 | 54.33 | 68.73 | **Yes** |

**Supplementary File 1. Statistical reporting of Chi-Squared T(x) values for comparing distribution differences in flow cytometry staining.** Significance was determined if Chi-Squared T(x) sample > Chi-Squared T(x) background.
