# Supplementary material for: Oxaliplatin resistance in colorectal cancer enhances TRAIL sensitivity via death receptor 4 upregulation and lipid raft localization
Source: eLife. 2021 Aug 3;10:e67750. doi: 10.7554/eLife.67750 (PMC8331188; doi:10.7554/eLife.67750)
Supplement: Transparent reporting form [file elife-67750-transrepform.docx]

***eLife’s* transparent reporting form**

We encourage authors to provide detailed information *within their submission* to facilitate the interpretation and replication of experiments. Authors can upload supporting documentation to indicate the use of appropriate reporting guidelines for health-related research (see [EQUATOR Network](http://www.equator-network.org/%20)), life science research (see the [BioSharing Information Resource](https://biosharing.org/)), or the [ARRIVE guidelines](http://www.plosbiology.org/article/info:doi/10.1371/journal.pbio.1000412) for reporting work involving animal research. Where applicable, authors should refer to any relevant reporting standards documents in this form.

If you have any questions, please consult our Journal Policies and/or contact us: [editorial@elifesciences.org](mailto:editorial@elifesciences.org).

**Sample-size estimation**

- You should state whether an appropriate sample size was computed when the study was being designed
- You should state the statistical method of sample size computation and any required assumptions
- If no explicit power analysis was used, you should describe how you decided what sample (replicate) size (number) to use

Please outline where this information can be found within the submission (e.g., sections or figure legends), or explain why this information doesn’t apply to your submission:

The number of patient samples utilized for this study was restricted by the number of patients who met the criteria for inclusion within the study (see Methods; Patient blood samples). Sample size was calculated using anticipated means from a previous study recently published by our lab (DOI: <https://doi.org/10.1186/s12885-020-07376-1>) using the “control” (90% CTC viability) and “TRAIL Liposome” (50% CTC viability) conditions. Standard deviation was estimated to be 25% with alpha=0.05 and power=80%. This gave a sample size of N=6 patients per group. Since blood samples were not split into control and treatment arms but used for both (i.e. blood was split to be treated by both controls and treatments), total minimum patient size was calculated to be N=6. The total number of patients enrolled was N=13, while total number of samples analyzed (including repeat patients follow-up draws) was N=21.

**Replicates**

- You should report how often each experiment was performed
- You should include a definition of biological versus technical replication
- The data obtained should be provided and sufficient information should be provided to indicate the number of independent biological and/or technical replicates
- If you encountered any outliers, you should describe how these were handled
- Criteria for exclusion/inclusion of data should be clearly stated
- High-throughput sequence data should be uploaded before submission, with a private link for reviewers provided (these are available from both GEO and ArrayExpress)

Please outline where this information can be found within the submission (e.g., sections or figure legends), or explain why this information doesn’t apply to your submission:

The number of replicates can be found in each figure legend, including the number of biological replicates “N” and technical replicates “n” if applicable. This is defined in first in the Figure 1 legend. The determination and exclusion of outliers for the image analysis studies are defined in “Methods; Confocal Microscopy and Image Analysis”. The number of total cells analyzed, n, after exclusion of outliers are specified within the figure legend.

**Statistical reporting**

- Statistical analysis methods should be described and justified
- Raw data should be presented in figures whenever informative to do so (typically when N per group is less than 10)
- For each experiment, you should identify the statistical tests used, exact values of N, definitions of center, methods of multiple test correction, and dispersion and precision measures (e.g., mean, median, SD, SEM, confidence intervals; and, for the major substantive results, a measure of effect size (e.g., Pearson's r, Cohen's d)
- Report exact p-values wherever possible alongside the summary statistics and 95% confidence intervals. These should be reported for all key questions and not only when the p-value is less than 0.05.

Please outline where this information can be found within the submission (e.g., sections or figure legends), or explain why this information doesn’t apply to your submission:

Figure legends contain information including: exact value of N, definition of error bars (SD or SEM), definition of p values, and statistical tests used. Raw data (individual values) are presented in all figures except for dose response curves and sensitization figures (calculated using the means from each group, see Methods; Annexin-V/PI Apoptosis Assay). Statistical methods used and definitions of significance are given in “Methods; Statistical Analysis”. Methods for calculating IC50 values are given within the figure legend (Figure 1 and Supplementary Figure 1).

(For large datasets, or papers with a very large number of statistical tests, you may upload a single table file with tests, Ns, etc., with reference to sections in the manuscript.)

**Group allocation**

- Indicate how samples were allocated into experimental groups (in the case of clinical studies, please specify allocation to treatment method); if randomization was used, please also state if restricted randomization was applied
- Indicate if masking was used during group allocation, data collection and/or data analysis

Please outline where this information can be found within the submission (e.g., sections or figure legends), or explain why this information doesn’t apply to your submission:

Criteria for designating patient blood samples as either oxaliplatin resistant or oxaliplatin sensitive are explained within the Figure 7 legend.

**Additional data files (“source data”)**

- We encourage you to upload relevant additional data files, such as numerical data that are represented as a graph in a figure, or as a summary table
- Where provided, these should be in the most useful format, and they can be uploaded as “Source data” files linked to a main figure or table
- Include model definition files including the full list of parameters used
- Include code used for data analysis (e.g., R, MatLab)
- Avoid stating that data files are “available upon request”

Please indicate the figures or tables for which source data files have been provided:

Raw data files for all figures as well as code used to analyze confocal images are uploaded to Dryad (doi:10.5061/dryad.3xsj3txg3). Please find the data set attached by using the following link: <https://datadryad.org/stash/share/1RpUbkyF5GkPu1Gxbct0hXyihLOR6vimO1N4IoOipaQ>
